# New 5-D Coding System for Categorization of Plastic Surgery Conditions

**DOI:** 10.29252/wjps.8.3.388

**Published:** 2019-09

**Authors:** Saurabh Gupta, Ravi Kumar Chittoria, Vinayak Chavan, Abhinav Aggarwal, Chirra Likhitha Reddy, Padmalakshami Bharathi Mohan, K. Shijina, Imran Pathan

**Affiliations:** Department of Plastic Surgery, Jawaharlal Institute of Postgraduate Education and Research (JIPMER), Puducherry, India

**Keywords:** Coding system, Plastic surgery, Categorization

## Abstract

**BACKGROUND:**

There is need for a coding system for categorizing the plastic surgery conditions to facilitate efficient data exchange, retrieval, research, time-series analysis, clinical audit, insurance and legal purposes. This is a pilot study to assess feasibility of newly proposed 5-D coding system in categorizing the plastic surgery conditions.

**METHODS:**

Retrospective analysis of records of plastic surgery patients visited in last 15 months was done. Each patient was assigned a code according to the newly proposed 5-D system of coding and recorded in excel sheet. Data analysis was done to categorize various plastic surgery conditions. Results of analysis were shown to 11 plastic surgeons and their feedback was taken.

**RESULTS:**

Feedback taken from participants showed 5-D coding system was useful and practically easy to categorize the plastic surgery conditions.

**CONCLUSION:**

Proposed new 5-D coding system is easy and useful in categorization of plastic surgery conditions.

## INTRODUCTION

Accurate and standardized coding system of plastic surgery conditions is important for reporting and analyzing medical data. Coding of the disease standardizes the documentation and makes it easy to identify, discuss, and retrieve the data. Coding facilitates the data exchange between hospitals and other medical related agencies. Coding also helps in efficient data retrieval, analysis and research. Further, it is useful for clinical audit, insurance and legal purposes.^1^^-^^5^ There are numerous coding systems existing for medical diseases and procedures.^6^^-^^8^

In India, there is no standard coding system followed by hospitals, database agencies and insurance companies. Also, there is no separate coding system for plastic surgery conditions. Plastic surgery is considered as a problem solving branch and the problems related to plastic surgery have been categorized into 5-Ds: Defect, Deformity, Dysfunction, Disability and Disfigurement.^9^ Authors have proposed a new coding system based on ‘5-Ds of plastic surgery’.^9^ This study highlights the role of newly proposed coding system in categorizing the plastic surgery conditions.

## MATERIAL AND METHODS

**Fig. 1:**
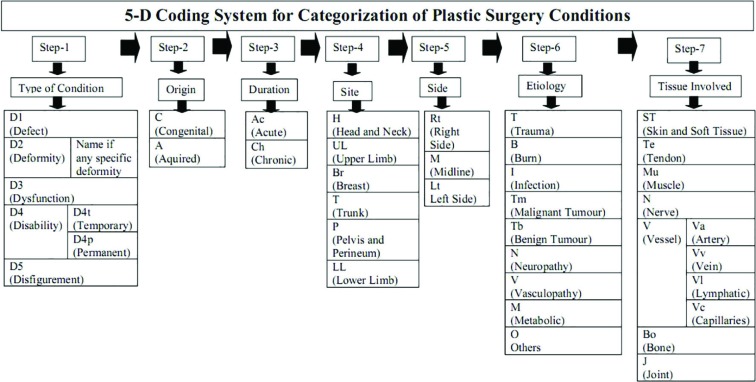
5-D coding system for categorization of plastic surgery conditions.

This study was done in Department of Plastic Surgery in a tertiary care hospital. This is a retrospective analytical study. Records of all patients who visited to one of units of the department during the period of January 2018 to March 2019 were studied. Based on diagnosis, each patient was assigned a code according to the 5-D system of coding (Figure 1). All codes were entered in an excel sheet and then analyzed for categorization of plastic surgery conditions. The results were presented to 11 plastic surgeons and their feedback was taken about the 5-D coding system.

5-D coding system involves 7 steps including Step 1: To assign first letter of code according to “Type of condition”. The types may be (i) *Defect*: means breach in tissue continuity with or without loss of tissue. It includes lacerations, wounds, ulcers, raw areas, cleft lip, cleft palate etc. (ii) *Deformity*: means alteration in shape and contour of the tissue. It may be specific deformity (eg. Swan neck, Boutonniere, halux valgus, springel deformity, etc.) or non-specific deformity. (iii) *Dysfunction*: means abnormality or impairment of functions. This includes motor, sensory or autonomic impairment due to nerve, tendon, muscle, bone, or joint pathology. 

(iv) *Disability*: means temporary or permanent loss of functions leading to limitation of day to day activities. Disability can be temporary or permanent. For example inability to walk due to tendon injury is a temporary disability, whereas leg amputation is a permanent disability. (v) *Disfigurement:* means aesthetically unacceptable surface abnormality that overlies normal contour. This includes unsightly scars, pigmentations, hair loss, etc. Depending upon the type of condition the first letter of the code is allotted as D1, D2, D3, D4t, D4p D5 or combination of any of these.

Step 2: To assign second letter of code according to ‘Origin of condition’. It can be either ‘C’ for congenital condition (present since birth) or ‘A’ for acquired condition. Step 3: To assign third letter of code according to ‘Duration of condition’. Any condition present since 3 months or less is considered as acute and denoted by letters ‘Ac’, whereas chronic conditions are denoted by letters ‘Ch’. Step 4: To assign fourth letter of code according to ‘Site of condition’. Site of condition is divided according to anatomical region. It may be ‘H’ for head and neck, ‘UL’ for upper limb, ‘Br’ for breast, ‘T’ for trunk, ‘P’ for pelvis and perineum and ‘LL’ for lower limb. 

Step 5: To assign fifth letter of code according to ‘Side of condition’. Side may be right side (Rt), left side (Lt) or midline (M). Step 6: To assign sixth letter of code according to ‘Etiology of condition’. It is categorized as Trauma (T), Burn (B), Infection (I), Benign Tumor (Tb), Malignant Tumor (Tm), Neuropathy (N), Vasculopathy (V), Metabolic (M) disorders, or Others (O). Step 7: To assign seventh letter of code according to ‘Tissue involved in condition’. It may be Soft Tissue and skin (ST), Tendon (Te), Muscle (M), Nerve (N), Vessel (Va for artery, Vv for vein, Vl for lymphatic, Vc for capillaries), Bone (Bo), or Joint (Jo). Finally the complete code of a plastic surgery condition consists of 7 letters separated by slash mark (‘/’). A condition can fall in multiple categories and thus its code may have multiple letters at some places.

## RESULTS

Totally, 377 patients were allocated codes for their plastic surgery conditions. These codes were presented in Table 1. Feedback responses of participants were presented in Table 2.

**Table 1:** Coding of plastic surgery conditions

**Serial number**	**Plastic surgery condition**	**Code allotted**	**No. of patients**
	Defect due to head and neck region malignancy	D1/A/Ac/H/-/Tm/ST, Mu, Bo	5
	Basal Cell Carcinoma (face)	D1/A/Ac/H/-/Tm/ST, Mu, Bo	6
	Maxillofacial injuries	D1, D2, D3/A/Ac/H/-/T/ST, Bo, J	12
	Post craniectomy calvarial defect	D1, D2/A/Ch/H/-/O/Bo	6
	Cleft lip	D1, D2/C/Ch/H/-/O/ST, Mu	8
	Microtia	D2/C/Ch/H/-/O/ST	2
	Robin-pierre syndrome	D2/C/Ch/H/-/O/ST, Bo	2
	Crouzon’s syndrome	D2/C/Ch/H/-/O/Bo	1
	TMJ ankylosis	D3/C/Ch/H/-/O/Jo	1
	Torticollis	D2, D3/C/Ch/H/-/O/Mu	1
	Hemifacial atrophy	D2, D5/A/Ch/H/-/ST, Bo	1
	Saddle Nose Deformity (post trauma)	D2 (saddle nose) /A/Ch/H/M/T/Bo	1
	Saddle Nose Deformity (post syphilis)	D2/A/Ch/H/M/I/Bo	1
	Post traumatic Nasal Deformity	D2/A/Ch/H/M/T/Bo	4
	Rhinophyma	D2/A/Ch/H/-/Tb/ST	1
	Neurofibroma (face)	D2/C/Ch/H/-/Tb/ST	2
	Ptosis (neurogenic acquired)	D3/A/Ch/H/-/N/N, Mu	1
	Post trauma facial palsy	D3/A/Ch/H/-/T/N	1
	Post trauma unsightly scar/mark over head and neck region	D5/A/Ch/H/-/T/ST	8
	Alopecia	D5/A/Ch/H/-/O/ST	4
	Wrinkles	D5/A/Ch/H/-/O/ST	3
	Unwanted facial Hairs	D5/A/Ch/H/-/O/ST	14
	Hairy Nevus cheek	D5/C/Ch/H/-/O/ST	4
	Tuberous breasts	D2/C/Ch/Br/-/O/ST	1
	Amastia	D2/C/Ch/Br/-/O/ST	1
	Poland syndrome	D2/C/Ch/Br/-/O/ST	1
	Malignant breast lump	D2/A/Ch/Br/-/Tm/ST	4
	Post Mastectomy Loss of Breast	D2/A/Ch/Br/-/Tm/ST	4
	Acute burns	D1/A/Ac/-/-/B/ST	26
	Post burn raw area	D1/A/Ch/-/-/B/ST	16
	Post burn contracture of axilla	D2/A/Ch/UL/-/B/ST	4
	Post burn contracture of fingers	D2/A/Ch/UL/-/B/ST	3
	Post burn contracture of neck	D2/A/Ch/H/-/B/ST	6
	Post Burn Ectropion	D2/A/Ch/H/-/B/ST	1
	Post burn microstomia	D2/A/Ch/H/-/B/ST	2
	Obesity related contour abnormality of trunk	D2/A/Ch/T/-/M/ST	8
	Congenital constriction ring syndrome hand	D2/C/Ch/UL/-/O/ST	3
	Syndactyly	D2/C/Ch/UL/-/O/ST, Bo	6
	Polydactly	D2/C/Ch/UL/-/O/ST, Bo	4
	Camptodactyly	D2/C/Ch/UL/-/O/ST, Bo	1
	Macrodactyly	D2/C/Ch/UL/-/O/ST, Bo	1
	Acute upper limb tendon injury	D3/A/Ac/UL/-/T/Te	35
	Closed metacarpal fractures	D3/A/Ac/UL/-/T/Bo	10
	Phalanx fractures	D3/A/Ac/UL/-/T/Bo	14
	Compartment syndrome	D3/A/Ac/UL/-/T/ST	4
	Degloving Injury hand	D1/A/Ac/UL/-/T/ST	3
	Finger Tip injuries	D1/A/Ac/UL/-/T/ST, Bo	50
	Squamous Cell Carcinoma (forearm)	D1/A/Ac/UL/-/Tm/ST	1
	Pyogenica Granulosum finger	D1/A/Ac/UL/-/Tb/ST	2
	Old upper limb tendon injury	D2, D3/A/Ch/UL/-/T/Te	14
	Chronic mallet finger	D2 (mallet), D3/A/Ch/UL/-/T/Te, Bo	4
	Swan neck deformity	D2 (swan neck), D3/A/Ch/UL/-/T/Te	2
	Post traumatic nail deformity	D2/A/Ch/UL/-/T/ST	8
	Vic	D2, D3/A/Ch/UL/-/O/Mu	1
	Brachial plexus injuries	D3/A/-/UL/-/T/N	4
	Ulnar claw	D2 (claw), D3/A/-/UL/-/T/N	2
	Median claw	D2 (claw), D3/A/-/UL/-/T/N	1
	Venous Malformation leg	D1, D2/C/Ch/LL/-/O/Vv	2
	Chronic venous ulcer of lower limb	D1/A/Ch/LL/-/V/Vv	6
	Acquired lymphoedema	D2/A/Ch/LL/-/O/ST, Vl	6
	Post trauma Foot Drop	D3/A/Ch/LL/-/T/N	2
	Tendo-achilis Shortening	D3/A/Ch/LL/-/T/Te	1
	Diabetic foot ulcer	D1/A/Ch/LL/-/M/ST	10
	Hansen’s Ulcer of Foot	D1/A/Ch/LL/-/I/ST	2
	Leg Defects with Both Bone Fractures	D1/A/Ac/LL/-/T/ST, Bo	12
	Soft tissue Sarcoma of leg	D1/A/Ac/LL/-/Tm/ST	4
	Hypospadias	D1/C/-/P/-/O/ST	8
	Vaginal atresia	D2/C/Ch/P/-/O/ST	3
Total			377

**Table 2:** Feedback responses of participants after analyzing 5-D coding system

**Question**	**Participants**										
	**1**	**2**	**3**	**4**	**5**	**6**	**7**	**8**	**9**	**10**	**11**
Do you feel that a coding system helps in data management?	Y	Y	Y	Y	Y	Y	Y	Y	Y	Y	Y
Is there any need for a separate coding system in plastic surgery?	Y	N	Y	Y	Y	N	Y	Y	Y	Y	Y
Does the 5-D coding system is helpful in categorizing the plastic surgery conditions?	Y	Y	Y	Y	Y	Y	Y	Y	Y	Y	Y
Does the 5-D coding system seem interesting to you?	Y	Y	Y	Y	Y	Y	Y	Y	Y	Y	Y
Did you find it easy to apply?	Y	N	Y	Y	N	N	Y	Y	Y	Y	Y
Did you find it comprehensive?	Y	Y	Y	Y	Y	Y	Y	Y	Y	Y	Y
Did you find it helpful in data retrieval and research?	Y	N	Y	Y	Y	N	Y	Y	Y	Y	Y
Did you find it helpful for insurance purposes?	N	N	N	N	N	N	N	N	N	N	N
Did you find it helpful in clinical purposes?	Y	N	Y	N	Y	Y	Y	N	Y	Y	Y
Does it provide a clinical guide to approach any plastic surgery problem?	Y	Y	Y	Y	Y	Y	Y	Y	Y	Y	Y
Will you use this coding system in your clinical practice?	Y	N	Y	N	Y	Y	Y	Y	Y	Y	Y
Will you use this coding system in record keeping of your patients?	Y	Y	Y	Y	Y	Y	Y	Y	Y	Y	Y
Do you feel that 5-D coding system needs modification and upgradation?	Y	Y	Y	Y	Y	Y	Y	Y	Y	Y	Y

Y=Yes; N=No

## DISCUSSION

Coding is an integral part of clinical data management. Senior author has already published categorization of plastic surgery conditions under 5-Ds.^9^ This coding system is based on 5-D categories. According to Ingenerf et al. standardized coding system provides ability of two or more systems or components to exchange information and to use the information that has been exchanged.^2^ A standardized coding system coordinates among different terminologies used for the similar conditions and thus facilitates data reuse for multiple purposes, with minimal transformations.^3^ One more advantage of coding system is that it enables accurate time-series analysis of individual patient.^4^

For example, if an individual visits multiple hospitals at different times for multiple problems, then analyzing his condition codes will provide information about time-line and treatment received for each condition, separately. At present, most of the countries use ICD-9 (International classification of diseases 9^th^ version), or ICD-10-CM (International classification of diseases 10^th^ version- clinical modification) for plastic surgery conditions; while OPSC-4 (Office of Population Censuses and Surveys Classification of Intervention and Procedures, version 4) or ICD-PCS (procedure coding system) for plastic surgery procedures.^1^^,^^6^^,^^7^

India is witnessing rapid spread of digital record keeping in healthcare system since last decade. It is prudent to use a standardized coding system to maximize beneficial utilization of this information bank. Our new proposed 5-D coding system for plastic surgery conditions can be a positive step towards this goal. First thing to be noted is that the 5-D coding system is not a replacement of diagnosis. It is a system of categorization of subjects with similar plastic surgery conditions. For example, this system will group together all congenital craniofacial abnormalities of head and neck region which involves soft tissue and bone. But, it will not be able to differentiate between cleft lip, cleft palate, and facial cleft. Similarly, it cannot differentiate between acute electric burn and thermal burn.

Another point is that a person can have multiple problems at multiple places in the body; thus there is no limit of letters in each section of the code. For example, a subject with diabetic foot ulcer with osteomyelitis of calcaneum, with swan neck deformity of multiple fingers due to rheumatoid arthritis will have a code: D1, D2 (swan neck), D3/A/Ch/UL, LL/any/M, I/ST, Bo, and J. Thus the subjects in different categories are overlapping. It is important to put correct letters under search for data retrieval and wisely derive true inference out of analysis. The 5-D coding system is comprehensive, but not exclusive. Same is pointed out by participants of our study also. 

All participants in our study found 5-D coding system interesting. Most of them agreed that it is easy to apply, reproducible and helpful in research. All the participants were ready to use this system in their patients’ record keeping. This system is not practical to be used for insurance purposes, as it is not based on management cost of the condition. All coding systems have limitation of being subjective at the point of data entry. Person who is allotting code should be well trained about the system and preferably should be having knowledge about the condition being entered. Same is true for 5-D coding system also, and code should be allotted by plastic surgery professionals only.

One positive aspect about 5-D coding system is that it is based on a clinical approach towards any plastic surgery problem. Thus it provides a guide to reach on a diagnosis; and it may be an important clinical education tool also. Cut off criteria of 3 months to categorize a condition as acute or chronic is arbitrary in 5-D coding system; and it can be different for different conditions. In etiology section numerous etiologies can be included. Authors have tried to restrict the coding to essential broad categories of etiology. Thus the code includes all metabolic conditions (diabetes, hypertension, dyslipidemia, rheumatoid arthritis etc.) in single category and cannot differentiate between them.

Certainly, there is a lot of scope of modification and upgradation in 5-D coding system as pointed out by participants of this study. This study is based on limited number of patients from a single centre. A large multicentric study is needed for validation of 5-D coding system. Newly proposed 5-D coding system is effective in categorization of plastic surgery problems. It is comprehensive and easy to apply. It is helpful in data retrieval and research purposes. It provides a clinical guide to approach any plastic surgery problem. This coding system is not a replacement of diagnosis and should be used as data attributes. It is not practical to be used for insurance purposes.

## CONFLICT OF INTEREST

The authors declare no conflict of interest.
